# Risk Factors for Liver Disease Cluster Geographically: A Precision Public Health Analysis of a UK City

**DOI:** 10.1111/apt.70088

**Published:** 2025-03-19

**Authors:** R. Parker, A. Taylor, R. Dukes, B. Wilks, A. Hinkson, D. Burn, I. A. Rowe

**Affiliations:** ^1^ Leeds Liver Unit St James's University Hospital, Leeds Teaching Hospital NHS Trust Leeds UK; ^2^ Leeds Institute of Medical Research at St James's, University of Leeds Leeds UK; ^3^ Leeds City Council Leeds UK; ^4^ Informatics Department Leeds Teaching Hospitals NHS Trust Leeds UK; ^5^ Leeds Institute for Data Analysis University of Leeds Leeds UK

**Keywords:** liver blood tests, liver disease, public health, Risk factors

## Abstract

These data describe the distribution of risk factors for liver disease in Leeds, a large city in the UK. Anonymised, unlinked data were aggregated to lower super output areas by the Leeds GP data extraction programme for deprivation, obesity, diabetes and alcohol use. Incident liver disease was quantified from coding of hospital admissions. Alcohol use, deprivation and obesity were associated with LD. Risk factors clustered together geographically. Liver blood tests were more frequently done in areas of low‐disease prevalence. These results illustrate health inequalities and support public health policies to reduce incident liver disease.

## Graphical abstract

Admissions to hospital due to liver disease are not distributed evenly across the city, but tend to cluster in particular areas that also tend to have high rates of deprivation, alcohol use, obesity and diabetes. This can guide public health efforts to prevent liver disease or initiatives to detect liver disease at an earlier stage.

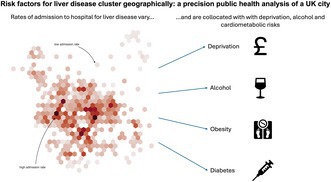

## 1 Introduction

Liver disease is highly prevalent and a common cause of premature deaths in the UK [1]. The majority of liver disease in the UK is associated with cardiometabolic factors [2] with or without hazardous alcohol use and could be preventable [3]. The prevalence of steatotic liver disease (SLD) is estimated at 27% in the UK, mainly due to metabolic dysfunction‐associated steatotic liver disease (MASLD, 91%) accounts for the majority of this burden, and alcohol‐related liver disease (ALD, 1%) and combined metabolic and alcohol‐related liver disease (metALD, 8%) account for other patients with SLD [4]. In terms of severe liver disease leading to hospital admission, alcohol is by far the largest single cause for this, where admissions due to ALD are equivalent to all other liver diseases combined [5]. Large‐scale mapping of liver disease in England has shown marked variation in rates of liver disease, where high rates of liver disease cluster in the northwest and northeast of the country [1]. In comparison, the distribution of dedicated hepatology services is not equitably distributed across England [6]. This mismatch is largely historical in origin but leaves many areas of high need without easy access to specialist clinical services. We examined the distribution of risk factors for liver disease in a large UK city using public health data at high resolution to accurately map risk factors for liver disease in terms of their geographical location and inter‐relationship.

## 2 Methods

### 2.1 Data

Leeds is a large city in the north of England with a population of approximately 812,000 people at the time of the last national UK census in 2021 (How life has changed in Leeds: Census 2021 [ons.gov.uk]). The majority of Leeds residents identify as ‘White’ in census data (79%), 9.7% identify as ‘Asian, Asian British or Asian Welsh’ and 5.6% identify as ‘Black, Black British, Black Welsh, Caribbean or African’. Routinely collected data were gathered for lower super output areas (LSOA) within Leeds. LSOA are geographical areas that contain approximately 1500 people and therefore vary in size depending on population density. In Leeds, the median adult population of an LSOA is 1287. Anonymised and unlinked data were aggregated to LSOA level by the Leeds GP data extraction programme regarding the following variables:

1. Deprivation index—This is a nationally used metric across England to indicate deprivation [7]. It is a single metric based on seven measures in each area: income, living environment, crime, barriers to housing and services, employment, health and disability, and education, skills and training. These are combined to generate a score where a higher number indicates a more deprived area.

2. Metabolic health

- Obesity: count of adults (age over 18 years) who have a BMI above 29.9 kg/m^2^.
- Diabetes: age‐standardised prevalence rates of diabetes in adults.

3. Alcohol

- AUDIT > 7: count of adults with an Alcohol Use Disorders Identification Test (AUDIT) score > 7.
- AUDIT > 16: count of adults with an AUDIT score of > 16.
- Alcohol licencing—off licence: the count of the number of licences for ‘off sales’, that is, shops selling alcohol for consumption elsewhere.
- Alcohol licencing—on licence: count of number of licences for ‘on sales’, that is, establishments selling alcohol for consumption on the premises for example pubs or restaurants.
- Referrals to alcohol services—the number of people in treatment for alcohol use disorder.
- Emergency alcohol admissions: emergency admissions to hospital due to conditions wholly or partly attributable to alcohol.

The SNOMED codes used to generate these variables are described through the UK Quality and Outcomes framework (https://digital.nhs.uk/data‐and‐information).

Leeds and the surrounding area are served by a single hospital system—Leeds Teaching Hospitals NHS Trust (LTHT), which provides laboratory services to primary care in Leeds. The following data were collected from LTHT records:

1. Severe liver disease is defined as admissions to hospital attributed to liver disease. These admissions were identified through clinical coding at LTHT using ICD‐10 codes K70.0 and cirrhosis codes I85.0, I85.9, I(i)98.2, I(i)98.3 for the calendar year 2019.
2. The number of liver blood tests (LBT) sent from primary care to the laboratory at LTHT.

### 2.2 Statistical Analysis and Data Visualisation

Non‐normally distributed variables were log transformed, and Poisson linear regression models were used to calculate incidence rate ratios (IRR) with 95% confidence intervals (CI) for association between risk factors and the count of liver‐related hospital admissions. Multiple measures of alcohol use were not included together in multivariable analysis, and separate analyses were performed to consider differing measures of alcohol use. Linear regression models were plotted, and the slope was tested to estimate the *r*‐squared value. To measure spatial colocation of risk factors for liver disease, Moran's I was calculated to quantify how closely values are clustered together. Moran's I ranges from −1 to 1, where −1 indicates that variables are perfectly dispersed, and 1 indicates that variables cluster perfectly together. A Moran's I of 0 indicates that a variable is randomly dispersed.

Geographical distribution of liver disease and risk factors was explored by mapping to LSOA. To anonymise areas within Leeds and to remove visualisation bias caused by densely populated LSOAs appearing smaller than rural LSOAs, each LSOA was plotted as a hexagon centred on the geographic coordinates of the LSOA (Figure S1). Each hexagon was shaded according to the prevalence of a selected risk factor. For this analysis, data regarding blood testing and liver admissions was normalised to the adult population of each LSOA by dividing the number of blood tests or liver‐related hospital admissions by the number of adults in each LSOA.

People living with end‐stage liver disease may have frequent admissions to hospital, for example, for abdominal drainage as a palliative procedure. This may lead to a single area having a disproportionately high number of admissions. To explore this, a sensitivity analysis was done where the count of individuals who were admitted to hospital with liver disease was considered rather than all liver‐related admissions. All analyses were undertaken in R using the gtsummary [8], ggplot [9] and spatialEco [10] packages.

### 2.3 Ethics

As this study used only anonymous, aggregate‐level data without additional study‐specific activity, individual consent and formal ethics approval was not sought.

## 3 Results

**FIGURE 1:**
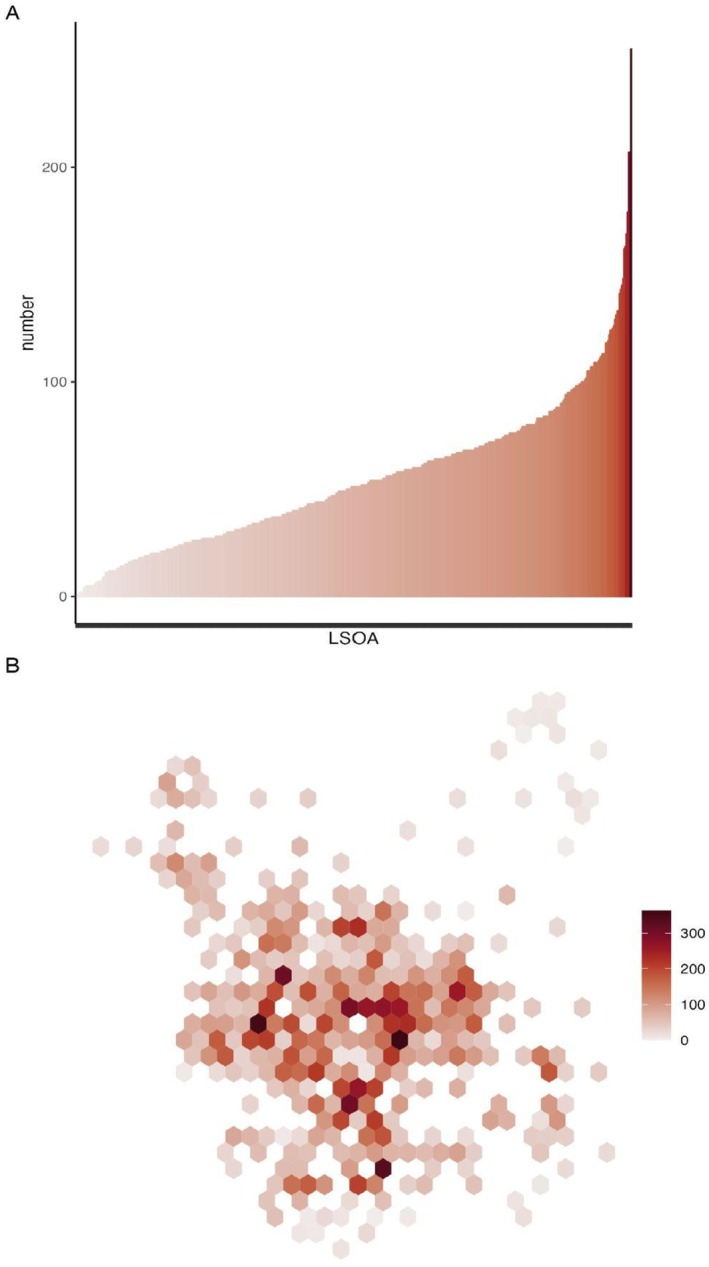
Admissions to hospital with liver disease across Leeds (A) variation in the number of liver‐related hospital admissions across all LSOAs (B) distribution of liver admissions across the city showing areas of high admissions (dark red) and lower frequency (lighter red).

**FIGURE 2:**
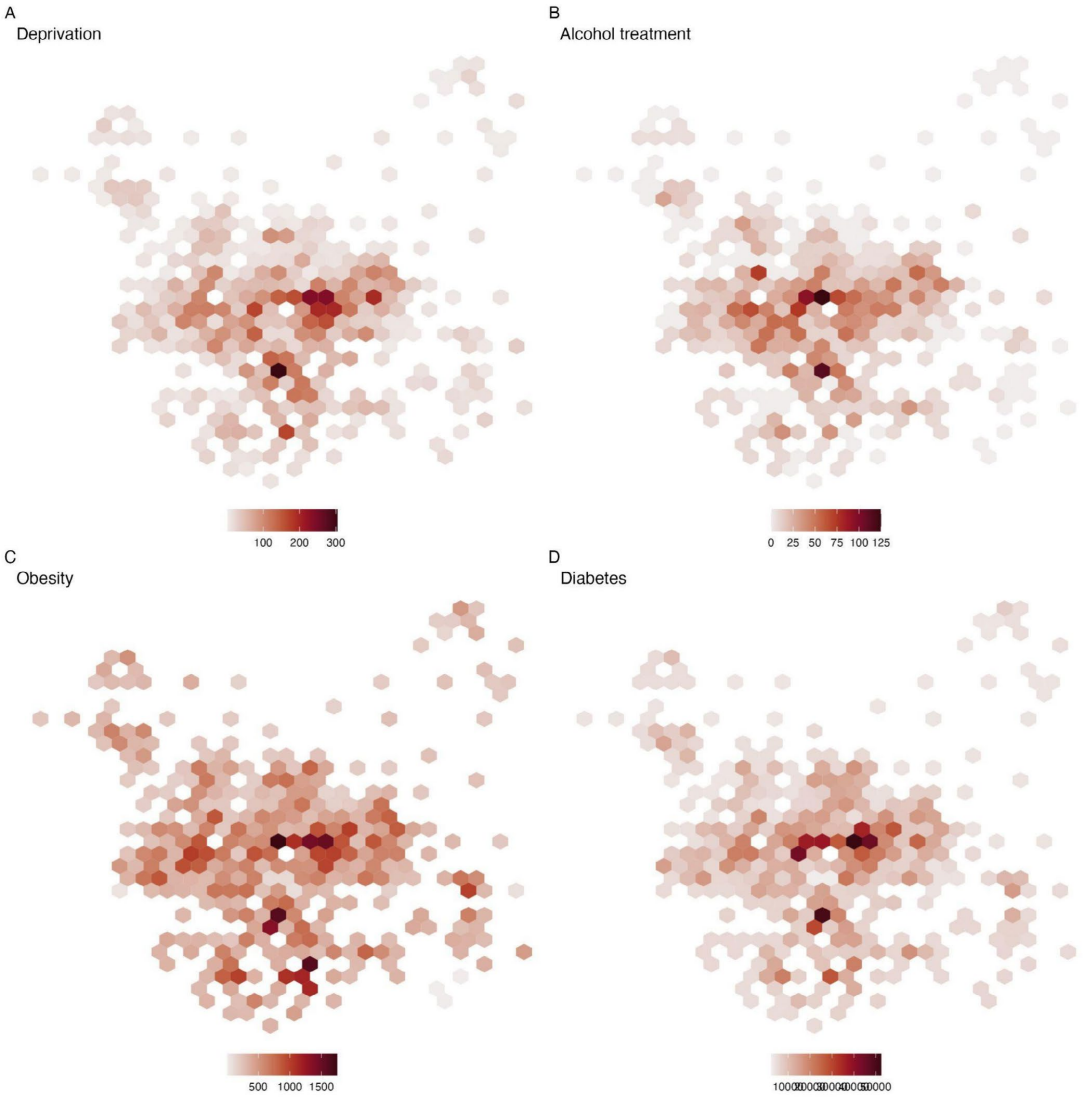
Distribution of risk factors for liver disease across LSOA in Leeds (A) Deprivation across Leeds, (B) count of people in alcohol treatment (C) count of persons with obesity (D) count of persons with diabetes.

Information was collated across 482 LSOA in Leeds. A total of 2219 admissions to hospital with liver disease were noted over the calendar year 2019. Over the same period, 190,809 LFT were sent from primary care. Liver‐related admissions to hospital varied significantly between LSOA (Figure 1). Liver admissions were not distributed evenly across the city but were clustered in particular areas (Figure 1B). Regression models showed that multiple factors were associated with liver disease where the statistically significant associations were seen between deprivation (IRR 1.57 (95% CI 1.55–1.59), *p* < 0.001), obesity (IRR 1.00, 1.18–1.26, *p* < 0.001) and diabetes (IRR 1.01, 1.00–1.01, *p* < 0.001) and counts of admissions to hospital (Table S1). Alcohol use was addressed by several different measures where both the count of individuals in alcohol treatment services and the count of individuals with an AUDIT score above 16 showed statistically significant associations with liver disease (IRR 1.03, 1.03–1.03), *p* < 0.001, and (IRR 1.18, 1.17–1.20) (Table S1). There was also a statistically significant association between admissions and the number of outlets licenced to sell alcohol (IRR 1.24, 1.22–1.27, *p* < 0.001). In all multivariable analyses, deprivation, obesity and diabetes were independently associated with liver admissions (Table S1). The count of persons with an AUDIT score above 16 (IRR 1.04, 1.03–1.06, *p* < 0.001) and the count of persons in alcohol treatment (IRR 1.01 1.01–1.01, *p* < 0.001) were independently associated with liver disease; however, the count of ‘off licence’ sales lost significance (Table S1). As the population size of each LSOA varied, we also included adult population size in a model where the same risk factors remained significant (Table S2). In sensitivity analyses, considering individuals with liver disease rather than all admissions, the same pattern of results was seen (Table S3, Figure S4). All risk factors for liver disease correlated with each other, where deprivation and alcohol treatment (*r* ^2^ 0.268, *p* < 0.001) and deprivation and obesity rates *r* ^2^ 0.331, *p* < 0.001 showed the strongest correlation (Table S1, Figure S2). Risk factors for liver disease were also co‐located, demonstrated by mapping and Moran's I (Figure 2, Table S2), indicating that risk factors for liver disease clustered together in the same populations. There was no association between the count of admissions from liver disease and the volume of LBT done in each LSOA (IRR 1.01, 0.98–1.03, *p* = 0.6). The geographic colocation of testing for liver disease and both risk factors was also weak (Table S4, Figure S3). When considering the collocation of liver blood testing and liver‐related hospital admissions, there was a negative relationship, that is, testing was more likely to take place in areas with low rates of hospital admissions (Moran's I −0.037, *p* < 0.001) (Figure S3).

## 4 Discussion

Liver disease is a common cause of premature death, especially amongst younger people of working age. Our data confirm the relationship between chronic liver disease, cardiometabolic risk factors and alcohol use on a population level. Using a precision public health approach to focus on the smallest unit available for analysis in England, we show that risk factors for liver disease are not equally distributed but cluster together in particular locations, which are also areas of high incidence of liver disease. Importantly, testing for liver disease did not focus on these areas despite a high overall volume of testing. Our findings illustrate health inequalities with regard to both the distribution of liver disease and the resources currently used to test for liver health.

Our data are specific to Leeds in the UK but have wider relevance. Leeds is a large city with a multiethnic population (How life has changed in Leeds: Census 2021 (ons.gov.uk)) The arrangement for testing for liver disease is probably typical for the UK, at least in 2018. An understanding of the local epidemiology of liver disease can help underpin a hepatology service. The arrangement of clinical services in Leeds with a single central laboratory and hepatology service allows confidence that we have captured the relevant data for analysis. There are watershed areas at the edges of our region that may send tests or referrals to different centres; these areas were removed from the analysis. We cannot be as certain about other aspects of our data; for example, the prevalence of people with high AUDIT scores relies on an accurate AUDIT being completed, which may not be the case in all individuals. This report also cannot comment on hepatitis B or C as drivers of liver disease, as information on the prevalence of these conditions is not available to LSOA level for analysis. However, we note that the prevalence of hepatitis B and C in the UK is estimated to be 0.12% and 0.14%, respectively, and these are therefore less likely to be major drivers of admissions to hospital in our city [11, 12]. Our data do not allow us to specify precisely which type of liver disease was driving admissions to hospital, and it should be noted that factors such as deprivation are associated with viral hepatitis as well as the other risk factors described in our manuscript.

Most cases of liver disease are associated with alcohol or cardiometabolic disease and are therefore preventable. Identification of the few people with significant liver disease among frequent risk factors requires targeted testing. Our data show how epidemiological data can be used to identify areas of high prevalence of disease and can support public health interventions and targeting of testing initiatives.

## 5 Data Sharing

Data used for this study may be shared with other investigators on application to the authors; it is likely that specific data‐sharing agreements will be required.

## Author Contributions

**R. Parker:** conceptualization, investigation, writing – original draft, methodology, visualization, writing – review and editing, formal analysis, data curation, project administration. **A. Taylor:** data curation, writing – review and editing. **R. Dukes:** data curation. **B. Wilks:** data curation. **A. Hinkson:** data curation, writing – review and editing. **D. Burn:** writing – review and editing. **I. A. Rowe:** conceptualization, writing – review and editing.

## Conflicts of Interest

The authors declare no conflicts of interest.

## Supporting information


Data S1.

## Data Availability

The data that support the findings of this study are available on request from the corresponding author. The data are not publicly available due to privacy or ethical restrictions.
