# Supplementary material for: Risk Factors for Liver Disease Cluster Geographically: A Precision Public Health Analysis of a UK City
Source: Aliment Pharmacol Ther. 2025 Mar 19;61(10):1697–702. doi: 10.1111/apt.70088 (PMC12013780; doi:10.1111/apt.70088)
Supplement: Supplementary file 1 — Data S1. [file APT-61-1697-s001.docx]

# Supplementary data

# Tables


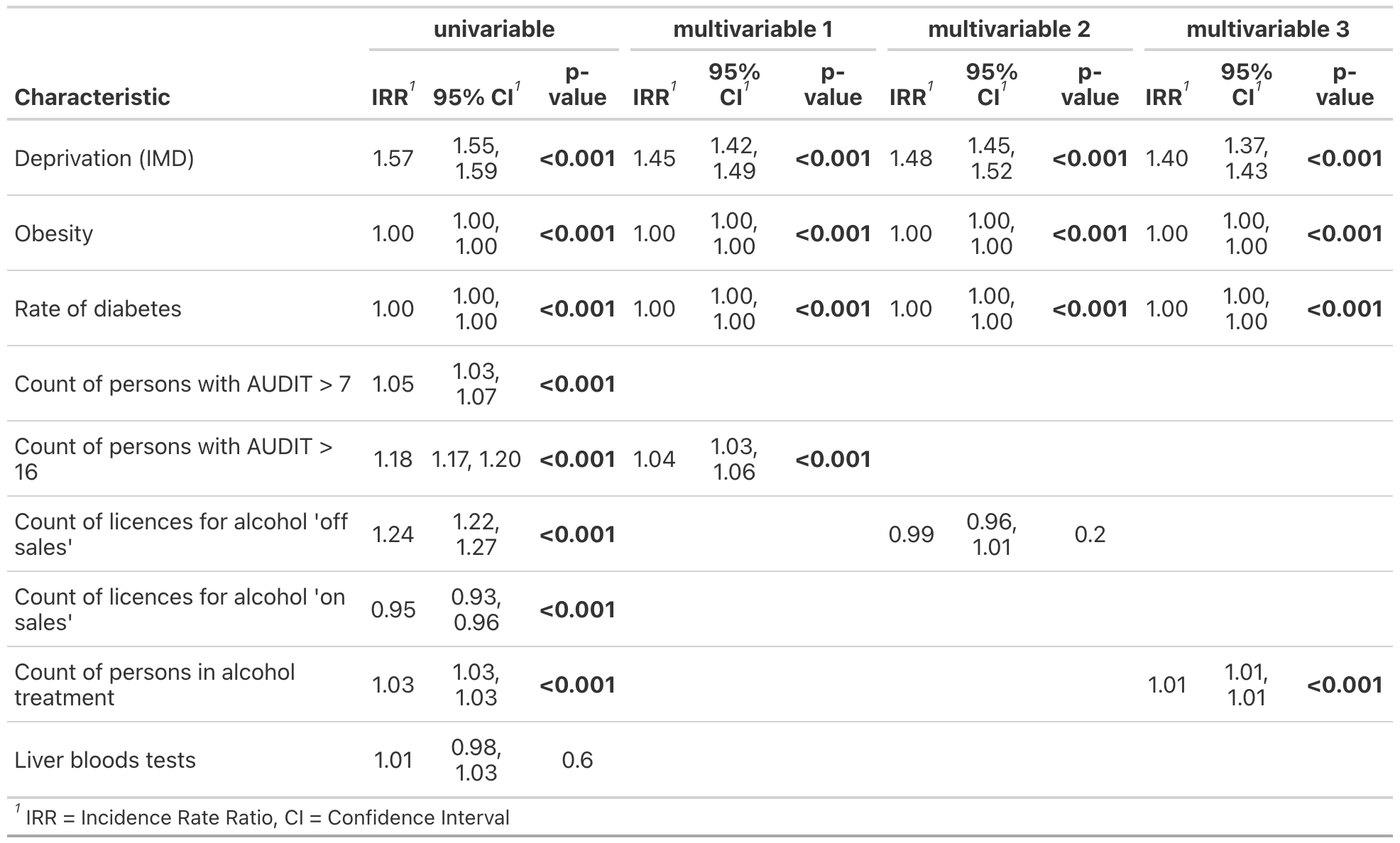


#### **Supplementary table 1**: linear regression to demonstrate association of risk factors for liver disease with admissions to hospital. IRR: incident rate ratio

####

####

#### **
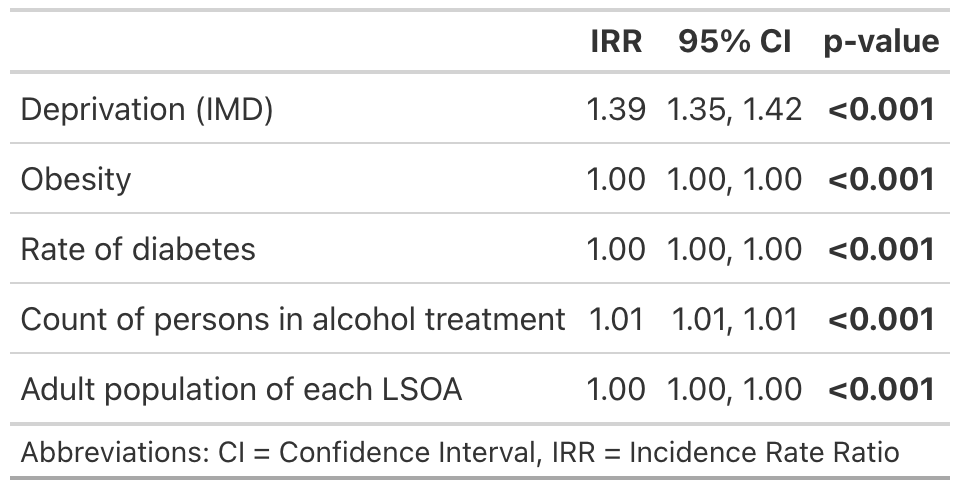
**

#### **Supplementary table 2**: linear regression to demonstrate association of risk factors for liver disease with admissions to hospital including population size of each LSOA. IRR: incident rate ratio

####

#### **
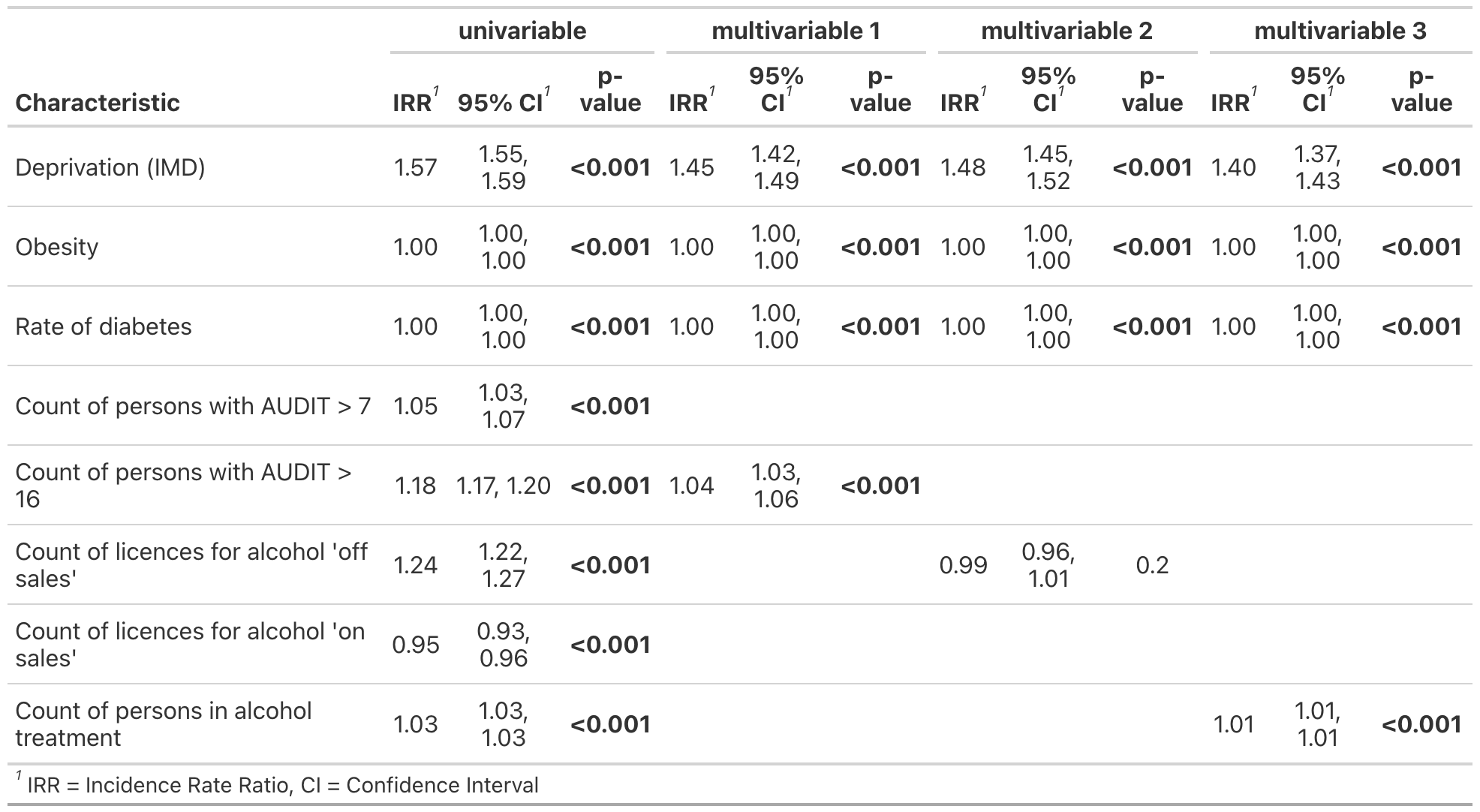
**

#### **Supplementary table 3**: linear regression to demonstrate association of risk factors for liver disease with individuals admitted to hospital. IRR: incident rate ratio

####

| **A: linear regression** | **Alcohol treatment** | | **Obesity** | | **Diabetes** | | **Liver blood tests** | |
| --- | --- | --- | --- | --- | --- | --- | --- | --- |
|  | R^2^ | p | R^2^ | p | R^2^ | p | R^2^ | p |
| Deprivation | 0.268 | <0.001 | 0.126 | <0.001 | 0.331 | <0.001 | 0.069 | <0.001 |
| Alcohol treatment |  | | 0.016 | 0.01 | 0.146 | <0.001 | 0.032 | <0.001 |
| Obesity |  | |  | | 0.065 | <0.001 | -0.001 | 0.44 |
| Diabetes |  | |  | |  | | 0.044 | <0.001 |
| **B: colocation** | Moran’s I | p | Moran’s I | p | Moran’s I | p | Moran’s I | p |
| Deprivation | 0.280 | <0.001 | 0.161 | <0.001 | 0.306 | <0.001 | -0.06 | <0.001 |
| Alcohol treatment |  | | 0.139 | <0.001 | 0.271 | <0.001 | -0.05 | <0.001 |
| Obesity |  | |  | | 0.162 | <0.001 | 0.001 | ns |
| Diabetes |  | |  | |  |  | -0.07 | ns |

#### **Supplementary table 4**: linear regression to demonstrate A: correlation between measures of deprivation and rates of alcohol treatment, obesity and diabetes in individual LSOA and B: measures of geographic colocation across the Leeds. The value for Moran’s I can range from -1 to 1 where -1 indicates that the variables of interest are perfectly dispersed, and 1: if the variables of interest are perfectly clustered together. A Moran’s I of 0 indicates that a variable of interest is randomly dispersed.

####

# Figures

#### **
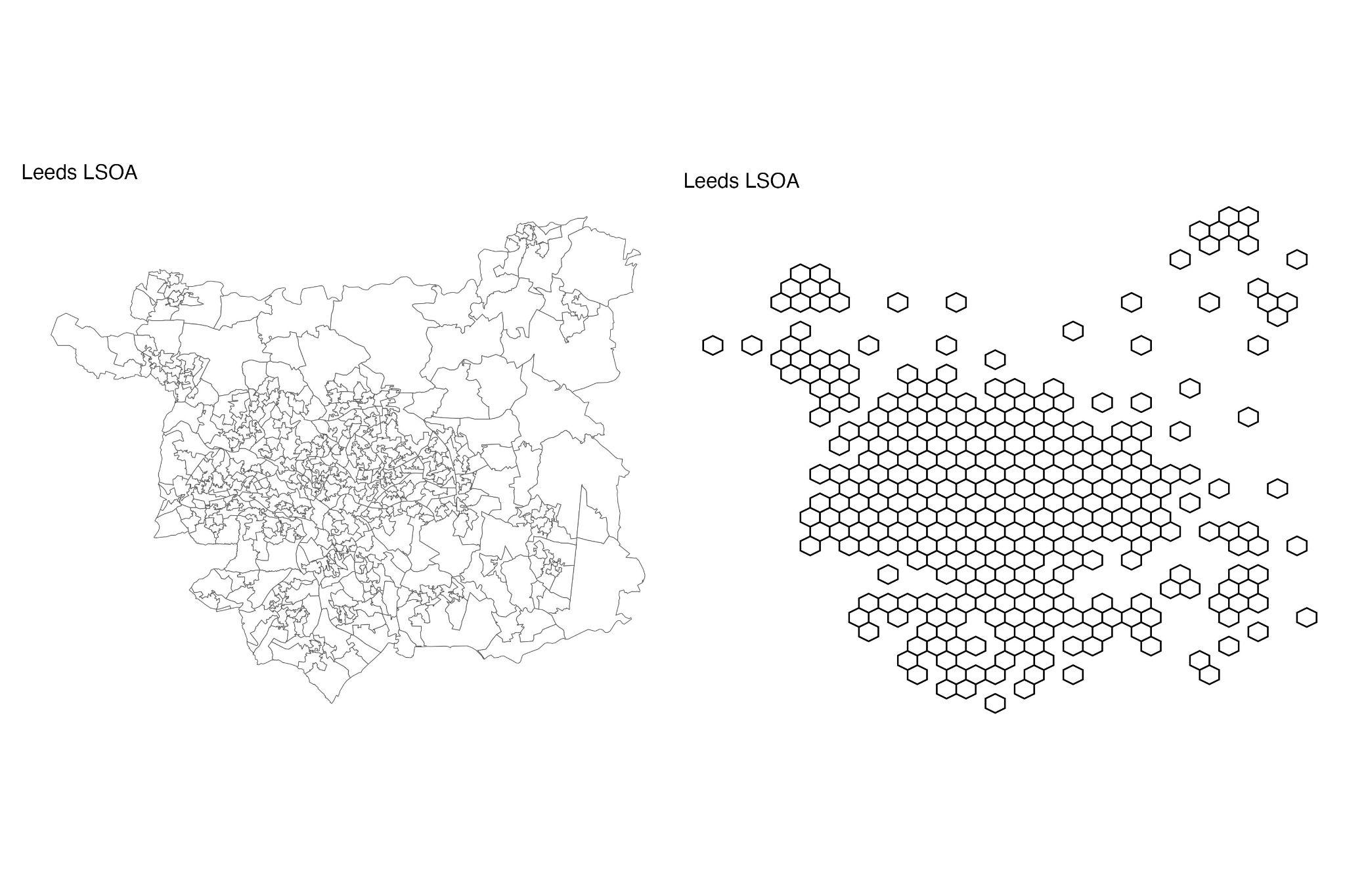
**

#### **Supplementary figure 1**: Leeds LSOA shown in **A** a standard map of Leeds and **B** represented as hexagon tiles to represent areas of similar population

####


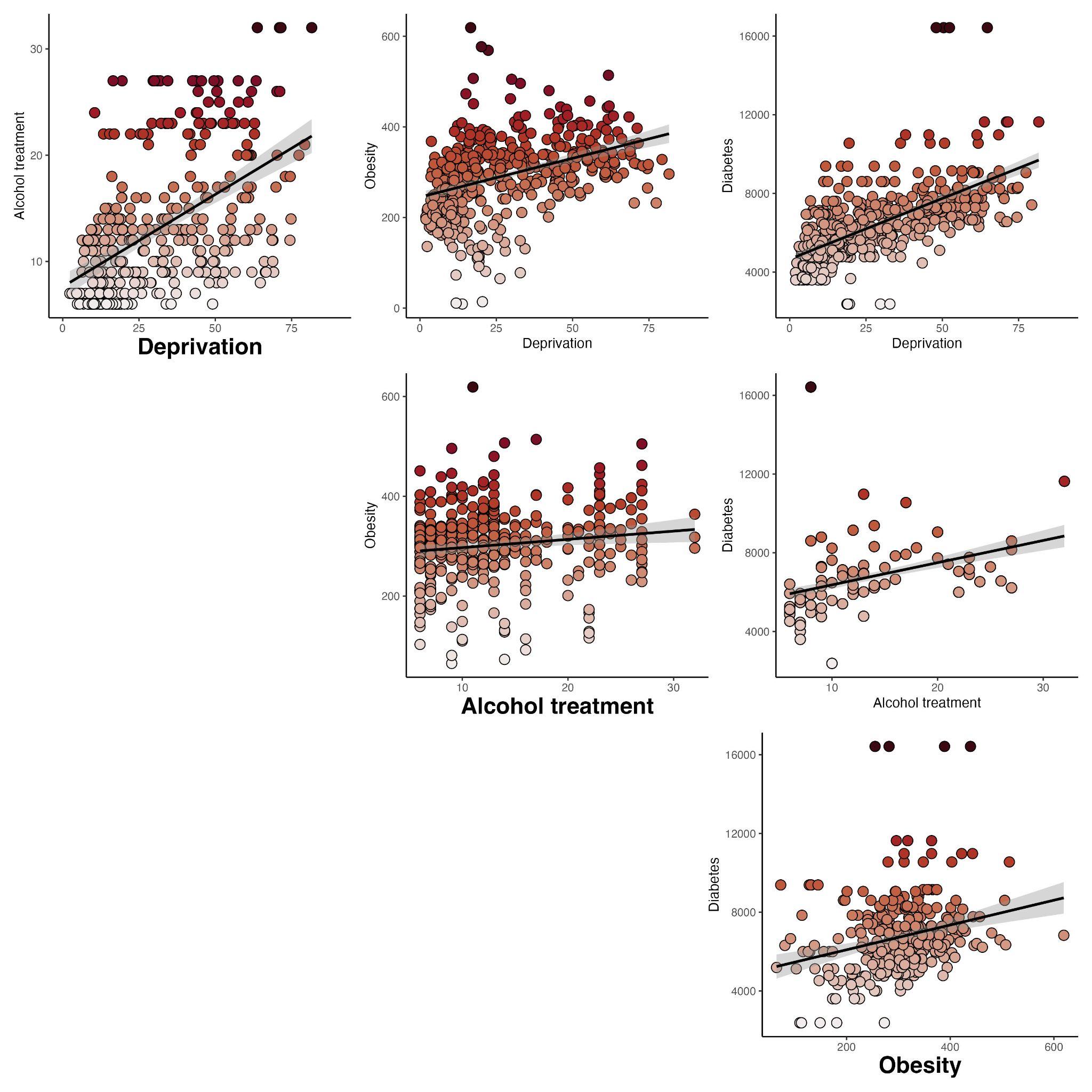


#### **Supplementary figure 2**: correlation between risk factors for liver disease in LSOA across the city of Leeds. Darker red circles indicate higher frequency of each risk factor.


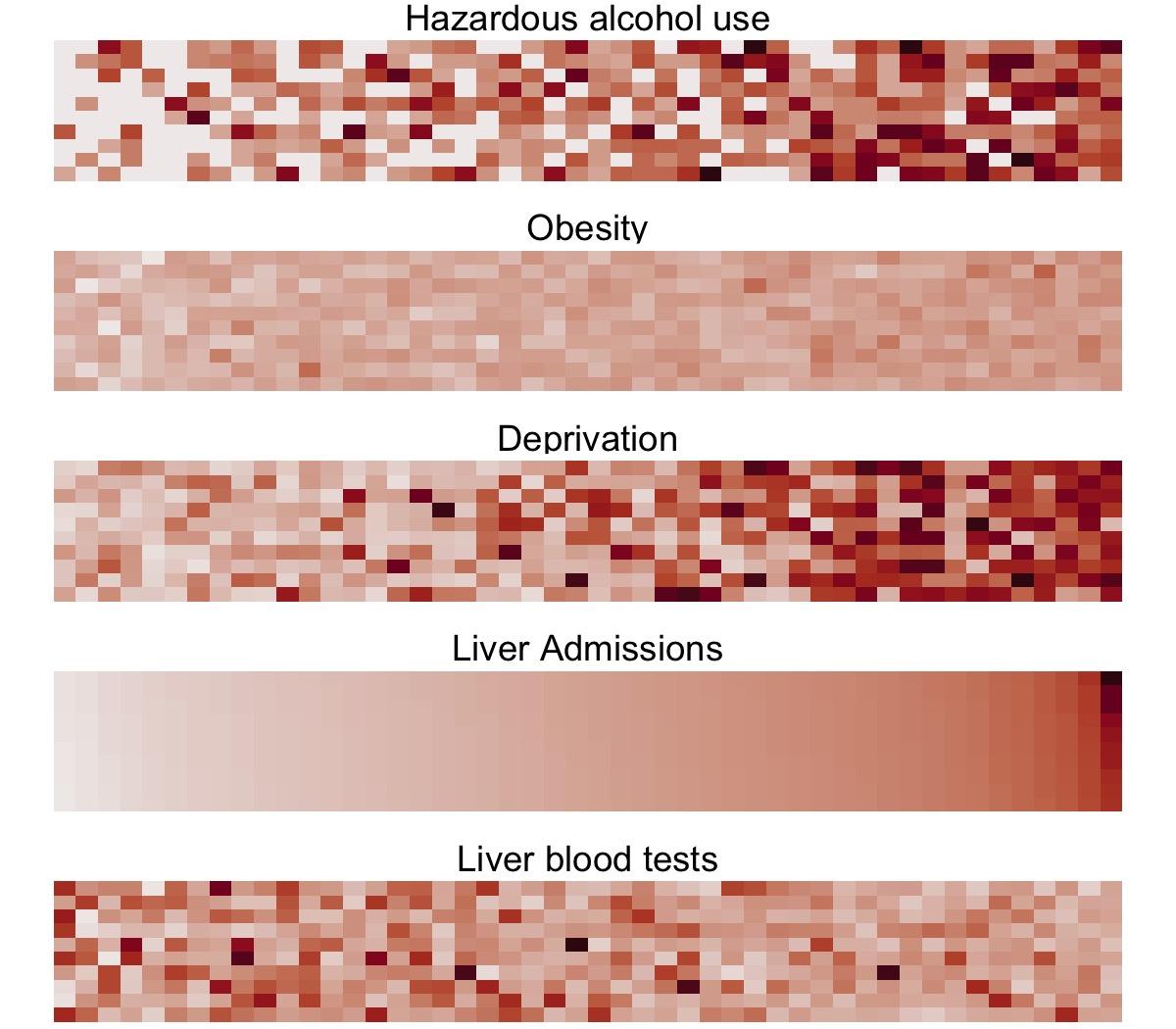


#### **Supplementary figure 3**: Each square represents an LSOA, and squares are ordered according to the count of liver admissions to demonstrate the relationship between risk factors, liver admissions and liver blood testing in Leeds.


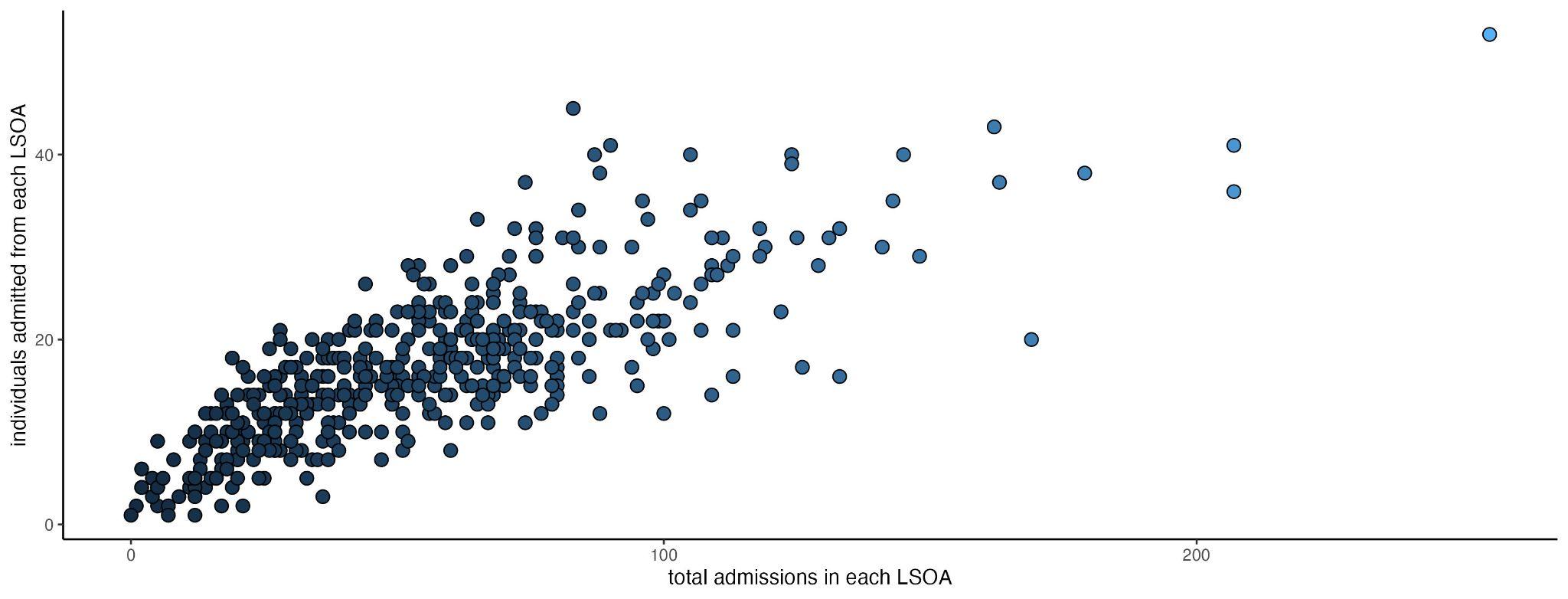


#### **Supplementary figure 4:** The number of individuals admitted from each LSOA and the overall number of admissions from each LSOA were strongly correlated (r^2^ 0.61, p<0.001)
